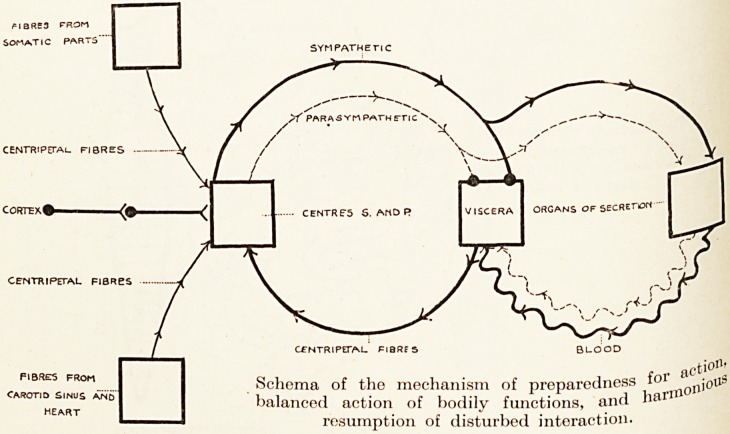# Surgery for Pain
*Extracted from a lecture delivered to the Bristol Medico-Chirurgical Society on 8th February, 1933.


**Published:** 1933

**Authors:** Ernest Rock Carling

**Affiliations:** Surgeon to Westminster Hospital


### The Bristol Medico-Chirurgical Journal

*" Scire est nescire, nisi id me*
*Scire alius sciret."*

SUMMER 1933.

### SURGERY FOR PAIN.*

BY

Ernest Rock Carling, F.R.C.S.,

*Surgeon to Westminster Hospital.*

---

The autonomic system plays so immensely important a part in the " corpore sano " that the " mens sana " could not exist without its healthy functioning; in fact, of these two separate parts it makes one indissoluble whole. Besides the part played by bodily feelings in the contexts out of which our psyche is built up, there is another aspect of psychological contexts and their mnemic evocation which we cannot touch upon now, bound up with " feeling-tone "; vague feelings of perfect and imperfect adaptation, suitability, aptness, fitness or unfitness, pleasure and unpleasure, upon which the proper or imperfect

* Extracted from a lecture delivered to the Bristol Medico-Chirurgical Society on 8th February, 1933.

functioning of the autonomic system has inevitably and unceasingly a potent influence.

With " Perceptions " we are not now concerned, but I must say a word or two of the emotions, which are " constituted by bodily sensations instinctively originated " ; the instincts being " primitive desires or inborn arrangements of the organism which lead it to respond to special situations in a special manner."

Instinctive responses to emotions such as " fear " and " rage " appear, at least in one aspect, as preparations for flight—*i.e.* escape—or for combat, and that preparedness is actuated through the autonomic system. Probably it is the same with other emotions such as " love " or " wonder," where " pleasure " takes the place of " unpleasure " or pain. The physiologist, who is only remotely concerned with pleasure and unpleasure, has also arrived at an interpretation of the functions of the autonomic system as serving preparedness for action ; Hess even regards *sleep* as such a function. He presents the autonomic system as organized not only to maintain a balance amidst all the changing and conflicting demands of the organs and tissues, but also to secure general re-invigoration of the organism as an interdependent whole, through sleep.

I want to direct your attention to an aspect of the autonomic system which is evoked by our present train of thought and has a philosophic connection with present-day surgery of the system. The separability of the two halves of the peripheral autonomic system, sympathetic and para-sympathetic, is to-day a commonplace. I would remind you that every efferent fibre, unlike the efferent somatic nerves, has a cell station upon its path, for the sympathetic in the para-vertebral ganglia—for the para-sympathetic in peripheral ganglia close to or in the tissues of the viscera. The effect of this is that an issuing impulse may be diffused over wide distribution, for pre-ganglionic fibres may influence, if not many cells in one ganglion, at least cells in many ganglia.

No doubt you are all familiar with the controversy over visceral pain, and are aware how little is known positively about the paths followed by afferent visceral impulses. None of us can doubt the existence of such impulses and paths, but experimental evidence is contradictory and conflicting. Afferent fibres, though they may travel in the interganglionic trunks, probably make no connections with cells of the para-vertebral ganglia—they are axons of cells in the spinal posterior root ganglia. It must be patent, therefore, that the removal of a ganglion, as such, will not affect the whole neurone liable to painful excitations—only in so far as the axons pass through the trunks and ganglia will the impulses be interrupted and the influence of ablation on the afferent will be likely to be far less extensive than that on the efferent field.

These diagrams are taken from Danielopolu to emphasize the endeavour of physiologists to abolish a common conception of the autonomic system as something isolated from the cerebro-spinal. It is suggested that the autonomic centres in bulb and cord are as subject to superior control as all other centres and cells therein ; that the subthalamic region represents a control, subject to cortical influence as we know, though the tracks of the connecting fibres have not been identified—a control that is not sympathetic or para-sympathetic, but as Danielopolu would say, amphotropic. He thinks not of opposed action of the two peripheral halves of the system, but of a tonus of

To illustrate the relation of the sympathetic and para-sympathetic, on the one hand to the centres in the hrain-stem, the basal ganglia and the cortex, and on the other hand to the glands of internal secretion, and tissues from which metabolites reach the blood-stream. To illustrate also that the neurones, of which the axons convey afferent impulses, belong to the spinal ganglia of the somatic system.

To illustrate the situation of the cell centres of pre-ganglionic fibres and the thalamic control of autonomic activity (*a*).

To illustrate the diverse paths of sympathetic and para-sympathetic fibers and their unifying centre in the bulb.

To illustrate sympathetic and para-sympathetic distribution to the gastro-intestinal tract.

To illustrate the essential part played by the internal secretions in completing and amplifying the function of the autonomic system; maintenance of the optimum environment for cellular and tissue activities in the face of all disturbing influences.

Schema of the mechanism of preparedness for action, balanced action of bodily functions, and harmonious resumption of disturbed interaction.

each which varies from individual to individual, and in each individual at times and seasons ; in health or ill-health. He looks, too, at the other extremity of the system, and there finds the potent chemical agents —adrenaline, choline, insulin and others as yet unidentified, some of them specific in their action upon the terminations of the system itself, some of them bound up with metabolism—as inhibitors on the one hand or, one might say, "catalysts" on the other hand of tissue activity.

Since my subject was first chosen a shower of papers on the sympathetic has descended upon us, and there is nothing in my experience unrecorded elsewhere. Upon a general review, I conclude that the element of success in my operations is concerned with pain, the pain for which the patient seeks relief. So far as that is relieved—*permanently* relieved—the patient is satisfied and the operator is justified. I have had ulcers healed, gangrene arrested, the texture of the skin improved, attacks of local syncope and asphyxia minimized, but the success has always been imperfect, and sometimes wholly or in part evanescent.

It is probable that painful visceral and vascular sensations are entirely functions of unstriped-muscular contractions or stretchings, whether in the walls of the arteries and arterioles, or of the hollow viscera and ducts, or involve nerves of somatic distribution to covering membranes and adjacent tissues.

In so far as we relieve pain, then, by removing para-vertebral ganglia and intermediate or connecting rami of the sympathetic (and there is abundant experimental evidence of the efficacy of these interruptions in the results of reported operations, especially for pain initiated in pelvic viscera) we relieve that pain partly, and perhaps largely, by cutting off *efferent* impulses, and that we know implies giving a free or freer rein to para-sympathetic stimuli. We are facilitating the washing away of accumulated histamin-like metabolites.

We know, too, that in respect to visceral distribution these two sets of opposing stimuli serve alternate functions; here the sympathetic is inhibitory, the para-sympathetic excitant—there the roles are reversed. Yet results seem to justify gross interference with what is normally a nicely-adjusted balance. Is there any theoretical or philosophic defence for such action? I think there is, and that it lies concealed in the very fundamentals of medicine.

I suppose that upon reflection all doctors will agree that by disease they mean departure from some standard of health. To define "health," as we have learnt, must be to chase a will-o'-the-wisp—it can only be defined for a given set of circumstances, for a given context, for a given age-period, for each several individual at a given moment.

It was Allbutt who put forward the idea, thirty-five years ago, that "health" is some stable relation between the whole of the bodily functions, activities and controls, and that after disturbance—by some mode of disease — a new stable state might be established in convalescence which differed in some respects as to the mutual relations of these functions and controls.

In our own day we have become so familiar with the term imbalance — for example, amongst the endocrine gland activities—and in our own field the autonomic system, that the relation of a stable, balanced state to "health" is an easily-grasped and even convincing or obvious idea. Moreover, we are so familiar with the idea of alternative stability in stereochemistry and particularly in the molecular structure of the chemical constituents of our bodies—and also in relation to the orbits of electrons in the atoms of radioactive elements, that Allbutt's suggestion is now more than ever likely to be fruitful, and certainly, in the restricted field of our ganglion operations, it fortifies us in the feeling that when we interfere grossly, as we do, in the mutual action of the two sides of the autonomic control, and limit our interference to those cases in which we can discern a disturbance of quiet functioning, an *un*stable state, we may permit a resumption of successful relations, not at the old level or pitch, but at a new one which can harmonize with all the other constituents of total stability and establish a new position of health. Restored health after modes of disease, such as typhoid fever or nephritis or encephalitis or any gross disorder from which recovery takes place, is probably on a plane of stability different from the former, but not necessarily worse or more vulnerable.

At any rate, the more complete our knowledge of what we do when we operate the greater the refinement we shall be able to introduce in our proceedings, and the more readily will a fresh stable state be reached. It is obvious, when one thinks of such gross interference as removal of the stomach or the spleen, or a lobe of a lung or the thyroid, that the inherent powers of adaptation are very great, and if we remember the lower forms of life, such, for example, as the planarian worm, with its chains of ganglia, and consider the reformation of an intact and whole worm from a random section, it is not astonishing that a good deal of disturbance of organs, tissues, controls and interdependent parts should be compatible with a different but a new mode of health.

One must not wait for or expect necessarily a series of named " diseases " for which ganglionectomy is *the* treatment. Whenever one is tempted to speak of " a disease " or to mistake the classificatory symbol of a mode of departure from health for an " entity," it is imperative to go back to the philosophy of our science before praying in aid the art of " healing."

Let me now turn for a moment to my method of dealing with intractable bodily pain originating in areas whose affected nerves reach the spinal cord—excluding, that is to say, the sensory cranial nerves. You are familiar with the operation of chordotomy, which involves section of the antero-lateral or spino-thalamic columns. This I have never done; it frightens me to think of identifying to a millimetre the required sector of the circumference of the cord, and then of dividing neither less nor more, to the precise depth necessary. The cord, at the bottom of a laminectomy wound is not an easy structure to deal with, and I have therefore sought a more facile method. In man all, or very nearly all, the fibres conveying painful stimuli decussate within a few segments, crossing in the anterior commissure. I suggest, therefore, a median division, entering the posterior sulcus, passing the central canal, and after dividing the commissure, stopping short of the anterior spinal artery which runs in the depth of the anterior fissure. If this division be made of three to three and a half inches extent it should sever all paths for pain entering at a given root level. I have performed it four times; once in the cervical region for intractable and intolerable pain due to a sarcoma infiltrating posterior roots, twice in the dorsal region for gastric crises of tabes, and once in the lumbar region for rectal and genital crises.

The result in one of the gastric crises cases seemed to the patient a complete success, for though crises, *i.e.* attacks of vomiting, which did not distress him, recurred, they were entirely painless. The cervical region case was satisfactory to this extent, that on coming round from his anæsthetic the man declared his freedom from pain was "like being in heaven," and that he remained pain-free to his death, some months later, from the progress of his sarcoma. The other two patients died; one forty-eight hours after the operation, without any indication beforehand of shock and without any evidence at necropsy of defect in the operation—the division was exactly median and there was no bleeding. The other case—the lumbar region case—died fourteen days after operation of broncho-pneumonia, but I was partly responsible, for though the wound had healed by first intention, a little pus was found on the body of one of the vertebræ exposed, and also, I am sorry to say, the division was not exactly median in its deeper part. I do not think I should make that error again, for in this region, where the cord is more mobile, additional precautions must be taken to ensure secure holding during the incision. Several tension threads should be inserted at short intervals in the dentate ligament and held with great care.

To lose two out of four cases is, of course, very serious, but it must be remembered that both these men had suffered for years from very severe pain, necessitating large doses of analgesics, and that both were drug sodden and very poor lives. Nevertheless, *qui s'excuse s'accuse.* I give you the facts, and in spite of them, urge those of you who have opportunity to give the method a trial. I have not published it, because upon four cases one cannot base an opinion of its merit, and it is rarely, indeed, that a general surgeon comes across cases that demand and justify so drastic a method.

The diagrams in this article are reproduced from D. Danielopolu in *La Pratique Médicale Illustrée* (G. Doin & Cie, Paris), to whom acknowledgment is due.

## Figures and Tables

**Figure f1:**
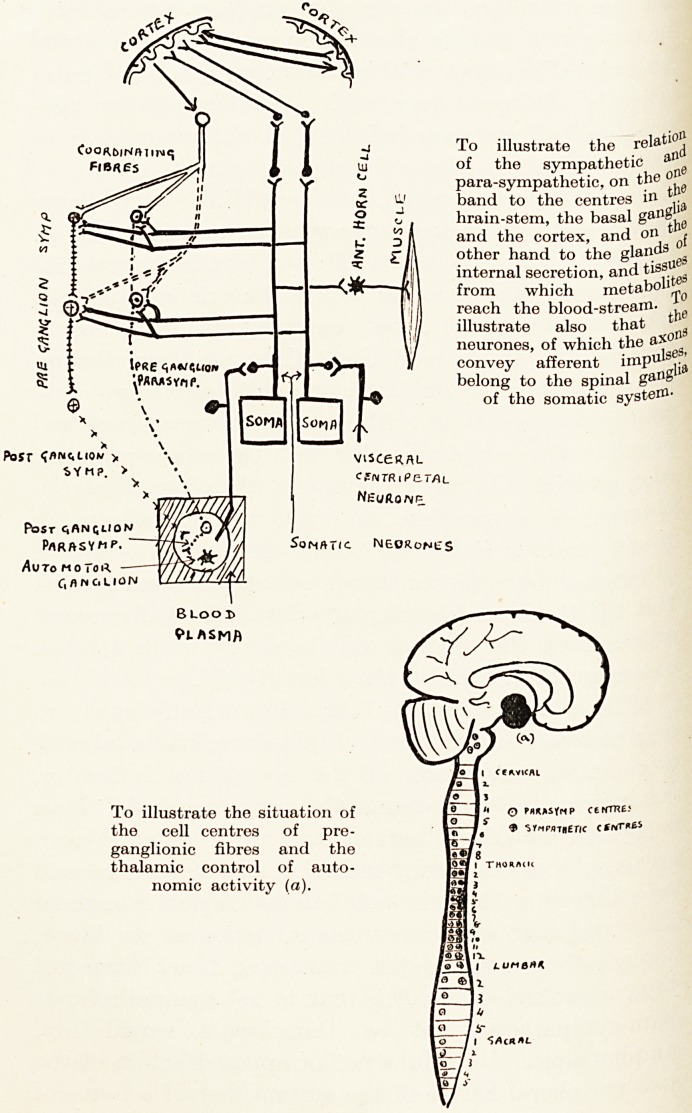


**Figure f2:**
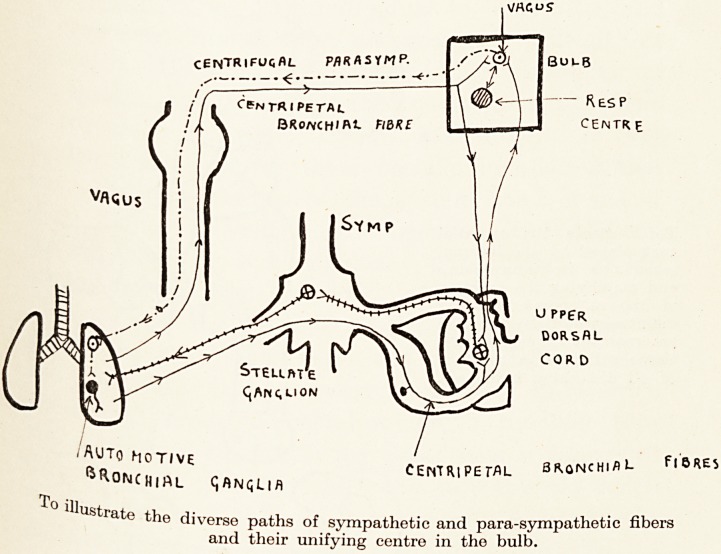


**Figure f3:**
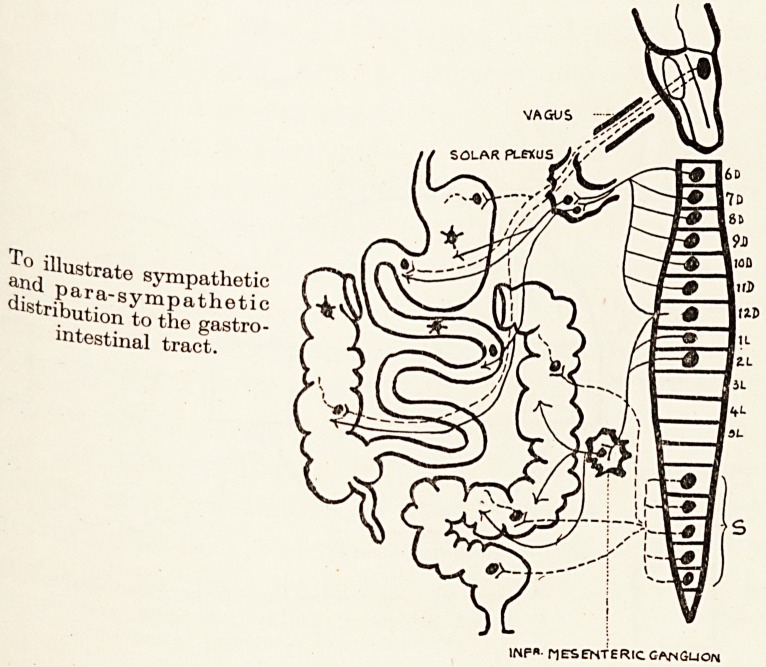


**Figure f4:**
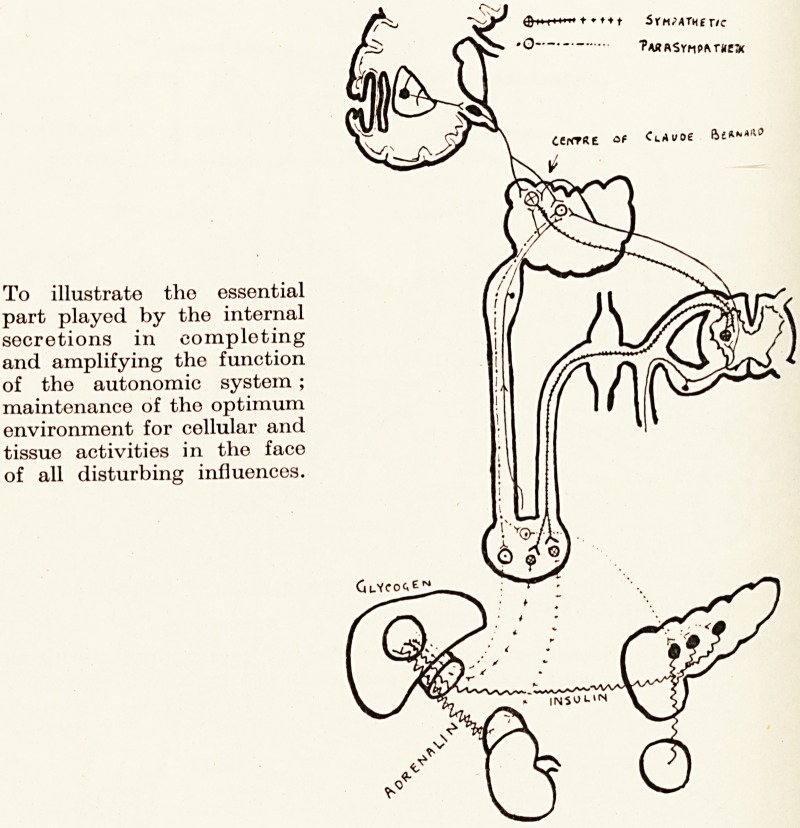


**Figure f5:**